# Limitations of the molybdenum blue method for phosphate quantification in the presence of organophosphonates

**DOI:** 10.1007/s00216-025-05850-y

**Published:** 2025-03-31

**Authors:** Ruoning Guo, Anna M. Röhnelt, Philipp R. Martin, Stefan B. Haderlein

**Affiliations:** 1 Geo- and Environmental Research Center, Department of Geosciences, Eberhard Karls Universität Tübingen, 72076 Tübingen, Germany; 2 Present Address: Division of Environmental Geosciences, Centre for Microbiology and Environmental Systems Science, University of Vienna, 1090 Vienna, Austria

**Keywords:** Aminopolyphosphonates, DTPMP, Glyphosate, UV–visible spectroscopy, Colorimetric analyses, Interference

## Abstract

Organophosphonates (OPs) are widely used as chelating agents in domestic and industrial applications. While regarded as hardly biodegradable, OPs can undergo abiotic transformation with phosphate (PO_4_^3−^) as a main transformation product. As some OPs are suspected precursors of glyphosate in surface waters, their environmental fate is of current interest. Due to analytical challenges posed by quantification of individual OPs, monitoring PO_4_^3−^ formation is a widely used proxy to monitor OP transformations. The molybdenum blue (MB) method, employing UV/Vis spectroscopy, is frequently used for PO_4_^3−^ quantification due to its sensitivity and operational simplicity. However, while interference of certain inorganic ions is well-documented, the effects of OPs on the accuracy of the MB method remain unexplored. This study investigated the effects of six OPs, namely *N*-(phosphonomethyl)glycine (glyphosate), 1-hydroxyethylidene(1,1-diphosphonic acid) (HEDP), iminodi(methylene phosphonate) (IDMP), aminotris(methylene phosphonate) (ATMP), ethylenediaminetetra(methylene phosphonate) (EDTMP), and diethylenetriaminepenta(methylene phosphonate) (DTPMP). Spectral analysis of pure PO_4_^3−^ standards using the MB method exhibits two characteristic absorption maxima (λ_max_) at 710 and 880 nm. In the presence of OPs, a new λ_max_ appears around 760 nm. This is accompanied by an increase in absorbance values at both 710 and 880 nm, leading to significant over-quantification of PO_4_^3−^ concentrations. Among the evaluated OPs, DTPMP exhibits the most substantial interference (PO_4_^3−^ over-quantification by up to 240%), while glyphosate causes minimal interference (≤ 20%). The effects are most pronounced at OPs:PO_4_^3−^ ratios ≥1. A case study simulating DTPMP transformation confirms PO_4_^3−^ over-quantification of up to 350%, revealing limitations of the MB method. Therefore, careful data evaluation and complementary analytical techniques for accurate PO_4_^3−^ measurements are indispensable in OP transformation research.

**Graphical abstract:**

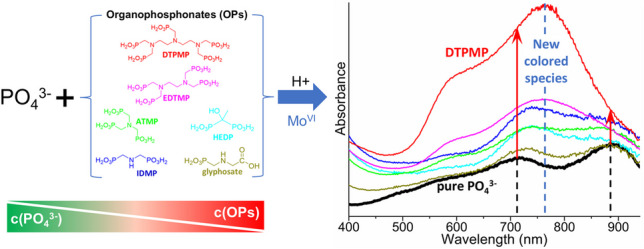

**Supplementary Information:**

The online version contains supplementary material available at 10.1007/s00216-025-05850-y.

## Introduction

Organophosphonates (OPs) are a class of organophosphorus compounds characterized by the presence of one or multiple phosphonic acid groups [–CH_2_–PO(OH)_2_]. Their physicochemical properties comprise high polarity and water solubility, thermal stability, and metal chelating ability [1, 2]. As structural analogues of well-known polycarboxylates such as ethylenediaminetetraacetate (EDTA), OPs are increasingly used as alternatives in industrial and consumer applications, for example, as complexing agents in textile and paper production, or as bleaching stabilizers in household detergents and cosmetics [3]. In 2012, OP consumption in Europe was reported to be 49,000 t, while global consumption reached 94,000 t [3]. Data on environmental concentration, however, is scarce. One of the few studies showed the total concentration of the five most commonly used OPs in the influent of wastewater treatment plants (WWTPs) ranged from 131 to 384 μg/L in 2019 [4]. A considerable amount of aminophosphonates is eliminated by sorption onto sewage sludge (around 85%) [5], but the remaining fraction in the effluent increases environmental phosphorus loading [6].

Recent research has shown that the transformation of diethylenetriaminepenta(methylene phosphonate) (DTPMP), one of the most widely used OPs in industrial applications, can lead to the formation of glyphosate [7], the world’s dominant herbicide, with yields ranging from 0.06 to 0.16 mol-% [8]. This discovery has significantly increased interest in the environmental fate of a wider range of OPs, particularly in relation to their transformation processes. Previous research has explored various mechanisms of OP transformations in laboratory experiments, including catalytic oxidation [9, 10], photolysis [11–13], hydrolysis [14–17], and microbial transformation [18]. Because analysis of individual OPs is challenging, quantification of one of their major transformation products, namely phosphate (PO_4_^3−^), is frequently used to prove and monitor OP degradation in laboratory studies. While various chromatographic techniques have been established for the separation and determination of PO_4_^3−^ species [19–21], UV/Vis spectroscopy, particularly the molybdenum blue (MB) method, is widely applied for PO_4_^3−^ determination due to its sensitivity and operational simplicity. The MB reaction occurs in two stages. Initially, a 12-heteropoly acid (12-MPA) is formed from PO_4_^3−^ and molybdate under acidic conditions. This intermediate is subsequently reduced to an intensely colored phosphomolybdenum blue (PMB) species (see Eqs. 1 and 2) [22].

1 $${\text{PO}}_{4}^{3-}+12{\text{MoO}}_{4}^{2-}+27{\text{H}}^{+}\to {\text{H}}_{3}{\text{PO}}_{4}{({\text{MoO}}_{3})}_{12}+12{\text{H}}_{2}\text{O}$$

2 $${\text{H}}_{3}\text{P}{\text{Mo}(\text{VI})}_{12}{\text{O}}_{40}+\text{Reductant}\to {{[\text{H}}_{4}\text{P}{\text{Mo}\left(\text{VI}\right)}_{8}{{\text{Mo}\left(\text{V}\right)}_{4}\text{O}}_{40}]}^{3-}(\text{PMB})$$

Since the establishment of the MB method [23], a number of modifications have been proposed to reduce the reaction time, prolong the stability of the PMB species, eliminate interferences, and increase the sensitivity of the method [24, 25]. Among these modified methods, the one by Murphy and Riley [26], which uses a mixed reagent containing ammonium molybdate, potassium antimony tartrate, sulfuric acid, and ascorbic acid, is the most widely accepted. Based on these developments, many countries and regional associations have established standardized protocols for PO_4_^3−^ determination. Table S1 in the Supporting Information provides a comparative analysis of the similarities and differences between these protocols.

Until today, the MB method remains a widely accepted and utilized approach for PO_4_^3−^quantification in laboratory OP transformation studies [27–30]. In previous research, the generation of PO_4_^3−^ has frequently been employed as a primary and often sole indicator to validate OP degradation in various contexts, such as hydrolysis [17], mineral oxidation [31], and biodegradation [32]. However, the effect of OPs on PO_4_^3−^ quantification by the MB method is still largely unexplored, despite the coexistence of OPs and PO_4_^3−^ in OP transformation studies. Nagul et al. [22] proposed that organic acids, such as tartrate and oxalate, inhibit the MB reaction by forming coordination complexes that sequester Mo(VI) and interfere with the formation of 12-MPA. Given the ability of OPs to coordinate with metals, they may cause similar interferences. While some studies have mentioned the interference of organic phosphorus compounds [33], they primarily refer to polyphosphates and labile organic P compounds, rather than OPs. When inaccuracies in PO_4_^3−^ quantification have been observed, they have been attributed to the hydrolysis of organic phosphorus compounds and thus the formation of PO_4_^3−^ during analytical procedures [34]. However, this explanation does not apply to scenarios involving OPs. For the quantification of total phosphorus using the MB method, a digestion procedure recommended in the established protocols is indispensable. However, when solely PO_4_^3−^ content is of interest–excluding any phosphorus derived from other molecules–digestion procedures cannot be applied. The effect of OPs on the accurate quantification of solely PO_4_^3−^ using the MB method is unknown. This issue is particularly critical in laboratory studies investigating the fate and transformation of OPs, where PO_4_^3−^ concentration serves as a proxy for OP transformation and therefore precise and accurate PO_4_^3−^ measurements are essential.

In this study, we investigated the effects of four commonly used OPs found in industrial products, namely aminotris(methylene phosphonate) (ATMP), ethylenediaminetetra(methylene phosphonate) (EDTMP), DTPMP, and 1-hydroxyethylidene(1,1-diphosphonic acid) (HEDP), along with iminodi(methylene phosphonate) (IDMP), a major transformation product of DTPMP, EDTMP, and ATMP [35], as well as glyphosate on the quantification of PO_4_^3−^ by the MB method. The MB method described by Laskov et al. [36] was employed to record full-range visible light absorption spectra (Vis-spectra) of PO_4_^3−^ standards in ultrapure water and compare them to PO_4_^3−^ spectra in OP matrices. To quantitatively evaluate the effects, single-factor experiments were performed by fixing either the PO_4_^3−^ or OP concentrations. Two absorption maxima (λ_max_) were identified from the Vis-spectra, allowing for quantitative comparison of absorbance values at λ_max_. Furthermore, PO_4_^3−^ formation from DTPMP transformation was simulated. This was done by varying the PO_4_^3−^:DTPMP ratios expected for different transformation stages. This study aims to provide guidelines for the use of the MB method for phosphate quantification in the presence of OPs, thereby preventing misinterpretations in OP transformation studies.

## Materials and methods

### Chemicals

Ammonium molybdate [(NH_4_)Mo_7_O_27_·4H_2_O] (≥ 99%) and potassium antimonyl tartrate [K(SbO)C_4_H_4_O_6_·0.5H_2_O] (99.0% ~ 103%) were purchased from Merck (Darmstadt, Germany). Potassium phosphate [K_2_HPO_4_] (≥ 99%), ascorbic acid [L( +)C_6_H_8_O_6_] (≥ 99%), and sulfuric acid [H_2_SO_4_] (96%) were purchased from Thermo Fisher Scientific (Waltham, Massachusetts, USA). Glyphosate (PESTANAL®, ≥ 98.0%), HEDP (≥ 95%), IDMP (≥ 97%), ATMP (≥ 97.0%) solids were purchased from Sigma-Aldrich (St Louis, MO, USA). EDTMP and DTPMP were bought as solid acid from Zschimmer & Schwarz (Lahnstein, Germany) under the name “CUBLEN ELC 950” and “CUBLEN D 900 GR,” respectively. In order to ascertain the purity of the purchased EDTMP and DTPMP, ^31^P-{^1^H}-NMR measurements were conducted. The analysis revealed the purity of EDTMP and DTPMP used in the experiments regarding P is > 97.48% and > 98.60%, respectively (for details, see the NMR section in Supporting Information). Additionally, phosphate impurities in the tested OPs were quantified using ion chromatography (IC) coupled to inductively coupled plasma mass spectrometry (ICP-MS). The analysis revealed that all measured phosphate concentrations were below the limit of detection (LOD, 0.0041 µM) or limit of quantification (LOQ, 0.0138 µM). Therefore, phosphate impurity levels are given as maximum values based on the LOD or LOQ, ranging from 0.2 to 2.7 mol-%. Further details can be found in the IC-ICP-MS section of Supporting Information. The water used for all solutions has been purified by an ultrapure water purification system (Barnstead, GenPurePro, Thermo Scientific, Waltham, MA, USA) down to a conductivity below 0.06 μS/cm^2^.

### Procedure for phosphate determination by molybdenum blue method

If not described differently, PO_4_^3−^ quantification was conducted using the MB method by Laskov (MB_Tü_) as outlined in the introduction. The molybdate sulfuric acid reagent stock solution contained 21.04 mM ammonium molybdate, 1.12 mM potassium antimony tartrate, and 4.60 M sulfuric acid. This reagent stock solution was stored in the dark at 4 °C until analysis.

The final coloring reagent contained 1.68 mM ammonium molybdate, 0.09 mM potassium antimony tartrate, 0.37 M sulfuric acid, and 0.01 M ascorbic acid. The ascorbic acid and therefore the final coloring reagent were freshly prepared on the day of analysis.

For color development, 2 mL of coloring reagent was mixed with 1 mL of either the sample or standard solution in 10-mm cuvettes (disposable plastic, BRAND, Wertheim, Germany). The cuvettes were sealed and shaken gently to ensure thorough mixing. After a reaction time of 30 min, the absorption spectrum was recorded over a wavelength range of 400 nm to 1100 nm using a UV–Vis spectrophotometer (METTLER TOLEDO, Greifensee, Switzerland).

## Results and discussions

### Determination of pure phosphate standards by the MB method

**Fig. 1:**
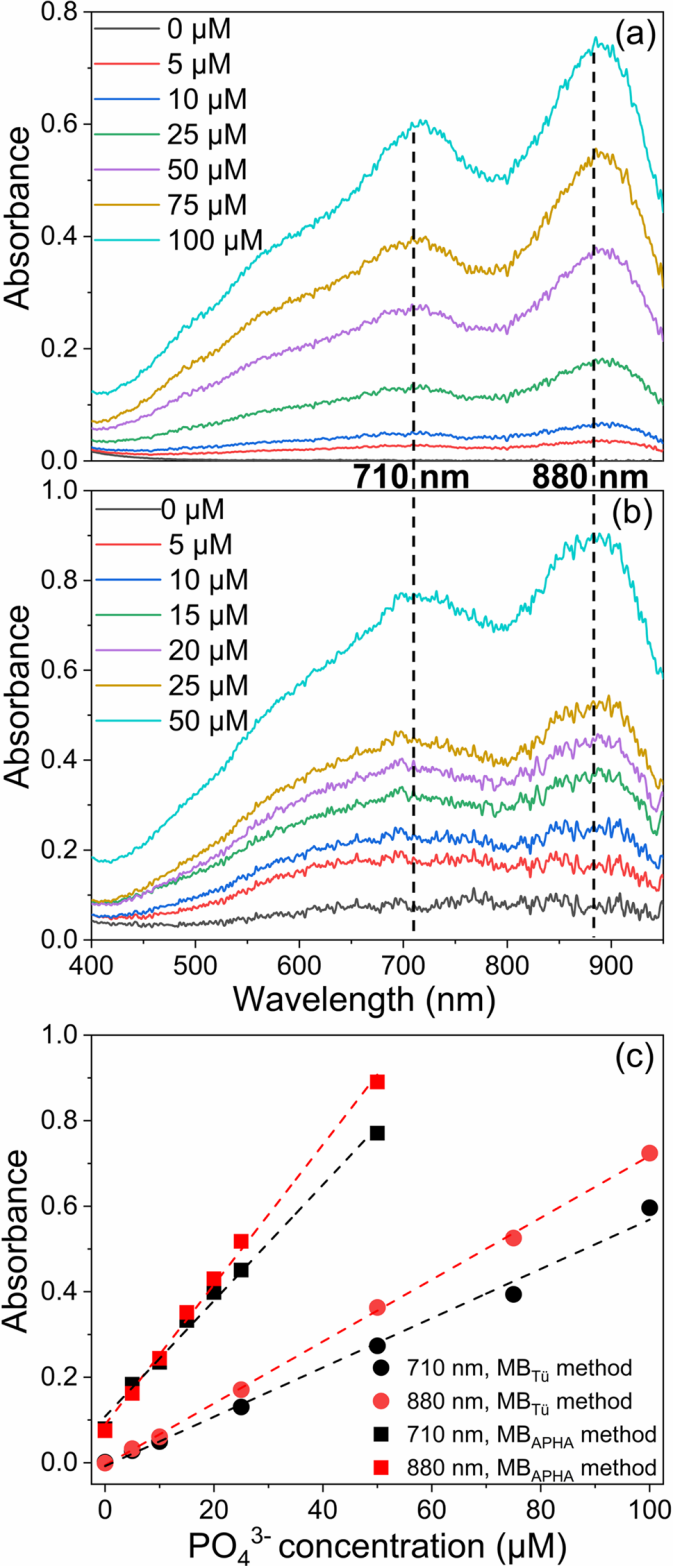
Absorption spectra of pure PO_4_^3−^ standards obtained using the **a** MB_Tü_ method and **b** MB_APHA_ method. **c** Calibration standards prepared using the MB_Tü_ method and MB_APHA_ method measured at λ_max_ = 710 nm and 880 nm

Pure PO_4_^3−^ standards were prepared in ultrapure water and subsequently analyzed using both the MB method according to Laskov et al. [36] employed in this study (referred to as MB_Tü_) and the most widely adopted protocol established by the American Public Health Association (MB_APHA_) [37]. The subtle differences between these two methods are detailed in Table S1. Given the differing concentration ranges suitable for linear analyses, a standard series of 5–100 µM was selected for the MB_Tü_ method, while a standard series of 5–50 µM was employed for the MB_APHA_ method. The absorption spectra exhibited a high degree of similarity (see Fig. 1a and b), suggesting both methods to be comparably effective for PO_4_^3−^ quantification. The two characteristic absorption maxima (λ_max_) at 710 and 880 nm, which indicate the formation of the PMB complex, were consistent and reproducible. Accordingly, the absorbance values at these two wavelengths are typically used for PO_4_^3−^ quantification. Additionally, external calibrations measured at 710 and 880 nm were compared for both MB variants (Fig. 1c). The coefficients of determination (R^2^) of all calibration curves, derived from linear regression, were ≥ 0.99, underscoring a high level of accuracy for both methods in quantifying PO_4_^3−^. The MB_Tü_ method was employed for all subsequent spectral investigations in this study.

### Effects of OP presence on PO_4_^3−^ quantification by the MB method

#### Qualitative effects of OP background on PO_4_^3−^ quantification

**Fig. 2:**
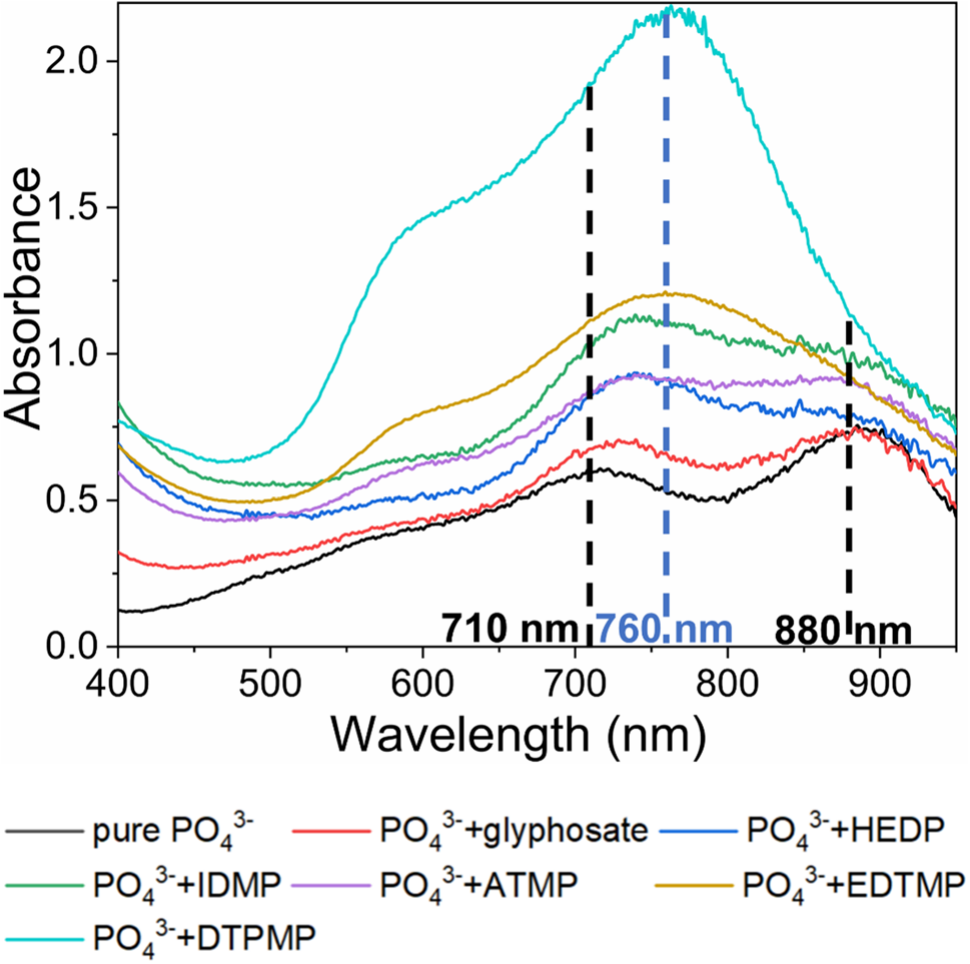
Comparison of absorption spectra for mix standards containing 100 µM PO_4_^3−^ and 1 mM of individual OPs

Following the validation of the MB_Tü_ method for pure phosphate standards, PO_4_^3−^ standards in the range from 5 to 100 µM were prepared in ultrapure water and in different OP matrices. The OP matrices contained one of the before mentioned OPs at a concentration of 1 mM (see Fig. S5). In the absorption spectra of standards containing either glyphosate or ATMP, the two λ_max_ at 710 nm and 880 nm remained unchanged (see Fig. 2). For the standards containing HEDP and IDMP, the λ_max_ at 880 nm was not discernible in the absorption spectrum. In the presence of EDTMP and DTPMP, a new λ_max_ at 760 nm emerged, which suggests the formation of new colored species. Furthermore, the absorbance values at λ_max_ = 760 nm showed a positive correlation with the PO_4_^3−^ concentration (see Fig. S5), indicating that PO_4_^3−^ is associated with the formation of this new species. Figure 2 shows the absorption spectra of 100 µM PO_4_^3−^ in ultrapure water compared to the spectra obtained in 1 mM OP matrices. In the presence of glyphosate, HEDP, ATMP, and IDMP, the λ_max_ at 710 nm exhibited a shift towards longer wavelength (up to 740 nm), with this shift occurring progressively in the order of the OPs listed. Simultaneously, the visibility of λ_max_ at 880 nm was compromised. With EDTMP or DTPMP in the matrix, a new λ_max_ at 760 nm obscured the original two λ_max_ = 710 and 880 nm. This indicates that the formation of the new species in the presence of OPs replaced the PMB species. Organic acids such as tartrate [38], oxalate [39], and malate [40] are known to inhibit PMB formation, which results from the formation of coordination complexes with Mo(VI) [41]. Similarly, the emergence of the λ_max_ at 760 nm caused by the presence of OPs is likely attributable to a coordination complex with Mo(VI).

#### Quantitative effects of OP background on PO_4_^3−^ quantification

**Fig. 3:**
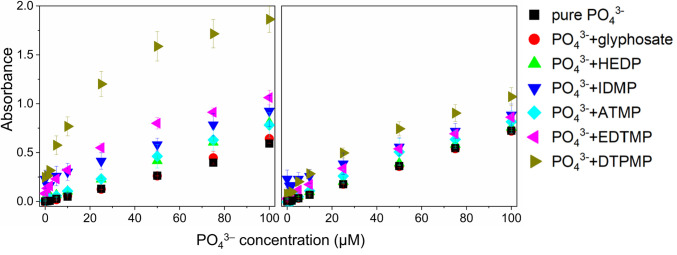
Absorbance at λ_max_ = 710 nm and 880 nm for mix standards with varying concentrations of PO_4_^3−^ and a fixed concentration of 1 mM OPs. Error bars represent the standard deviation of duplicate experiments. If error bars are not visible, their size is smaller than the marker used to represent the data

To quantitatively explore the effects of the six OPs on PO_4_^3−^ quantification, the absorption spectra of mix standards with varying concentrations of PO_4_^3−^ and 1 mM of each OP, respectively, were acquired (Fig. S5). The absorbance values at λ_max_ = 710 and 880 nm were further measured in duplicate (Fig. 3). The relative increase in absorbance for the different OP matrices relative to PO_4_^3−^ standards in ultrapure water was then calculated (Table S3). For all PO_4_^3−^ concentrations tested, the presence of 1 mM OP resulted in an increase in absorbance at both λ_max_, except for glyphosate. This increase was more pronounced at 710 nm. However, careful examination of the absorption spectra presented in Fig. S5 reveals that the observed increase in absorbances is not due to the enhancement of distinct absorption maxima at 710 or 880 nm for PMB species. Rather, the spectral evolution is characterized by the emergence of a new absorption band centered at 760 nm, which progressively increases in intensity. This newly developed λ_max_ partially overlaps the 710 and 880 nm regions, causing an apparent increase in absorbances at both wavelengths. Consequently, without a thorough analysis of the complete spectra for each measurement, one might erroneously conclude that the specific absorbance at λ_max_ of 710 or 880 nm has increased. The most significant increase was observed for DTPMP, with a relative 19.8-fold increase in absorbance at 710 nm, in a mixture containing 5 µM PO_4_^3−^ and 1 mM DTPMP.

Notably, PO_4_^3−^ impurities in OPs cannot account for the observed PO_4_^3−^ over-quantification in our study. This conclusion is based on two key observations. Firstly, nuclear magnetic resonance (NMR) analyses (Figs. S1–S3) revealed that phosphorus-containing impurities in the used EDTMP and DTPMP chemicals were minimal, amounting to only 1.37% and 3.40%, respectively. Even when assuming the highest possible PO_4_^3−^ impurities in OPs, as determined by the LODs of IC-ICP-MS, the contribution is quantitatively insufficient to explain the magnitude of over-quantification. For instance, in the case of DTPMP, the maximum possible impurity of 2.0 mol-% would only result in a fivefold increase in absorbance, which falls far short of the observed 19.8-fold increase. Secondly, if PO_4_^3−^ impurities were the sole cause, we would expect to see a linear relationship between absorbance and PO_4_^3−^ concentration, with a constant offset relative to pure PO_4_^3−^ standards. However, our results, as demonstrated in Fig. 3, showed spectral changes that are inconsistent with this explanation. Collectively, these findings indicate that the formation of new species rather than simple additive effects from impurities is the reason for the observed over-quantification of PO_4_^3−^.

Given that the fixed concentration of OPs with varying PO_4_^3−^ concentrations induced distinct quantitative effects on the absorbance values at 710 and 880 nm, it seemed conceivable that varying concentrations of OPs would lead to additional effects. The systematic evaluation of the effects of different OPs and PO_4_^3−^ concentrations will be discussed in the following.

### Impact of varying OP concentrations

**Fig. 4:**
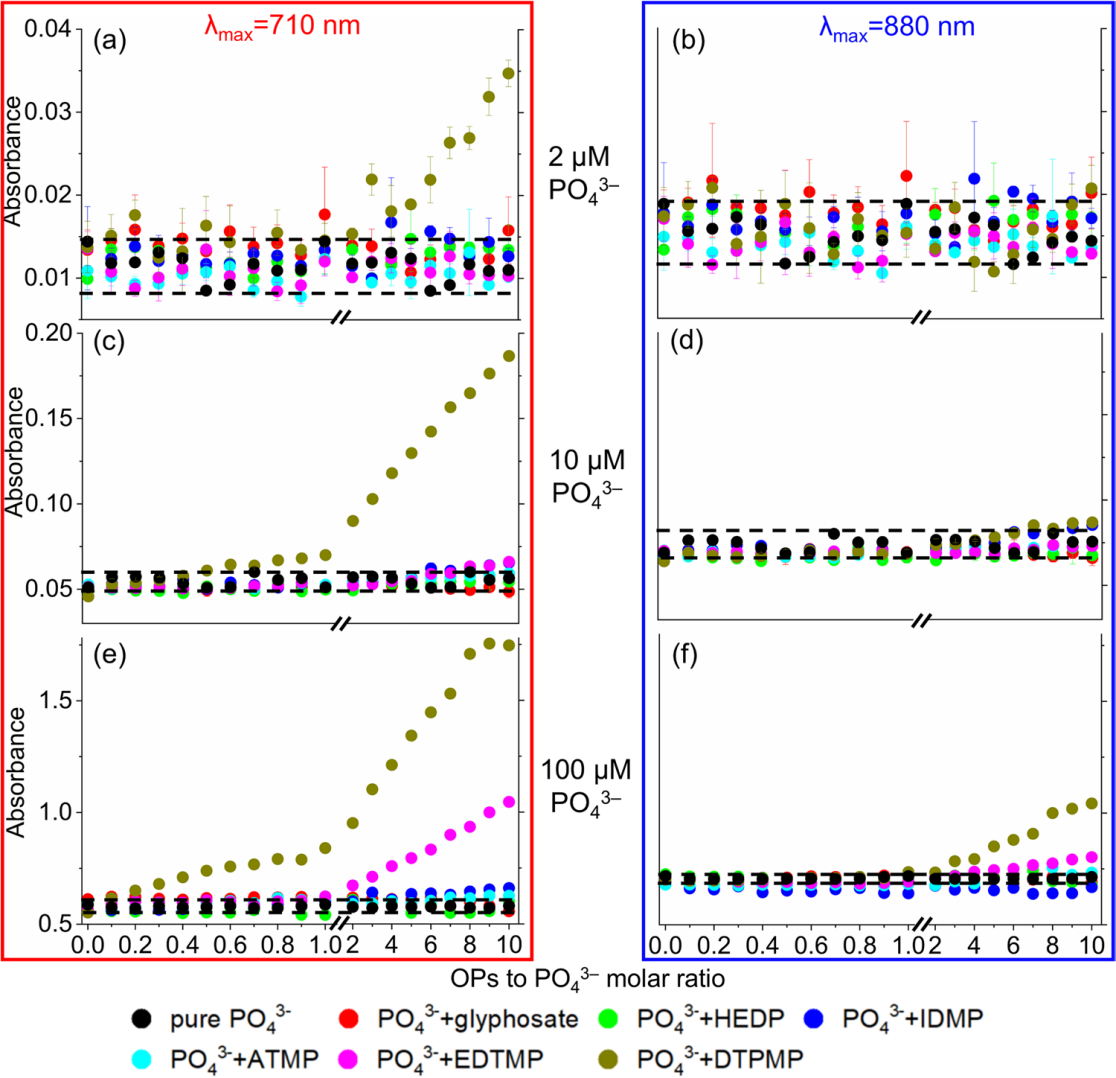
Absorbance at λ_max_ = 710 nm (left column) and 880 nm (right column) for fixed PO_4_^3−^ concentrations of 2 µM, 10 µM, and 100 µM, with varying OPs-to-PO_4_^3−^ molar ratios, using the MB_Tü_ method. The dotted lines represent the standard deviation of ten individual PO_4_^3−^ standards at three fixed concentrations, with all SD values < 0.005. Columns share the same x-axis, and rows share the same y-axis. Error bars represent the standard deviation of triplicate experiments. If error bars are not visible, their size is smaller than the marker used to represent the data

To investigate the effects of varying OP concentrations on PO_4_^3−^ quantification, PO_4_^3−^ standards at three different concentrations (2 µM, 10 µM, and 100 µM) were prepared containing varying OP concentrations (0.2–20 µM, 1–100 µM, and 10–1000 µM). While previous discussions have primarily focused on relatively high molar ratios of OPs:PO_4_^3−^ (≥ 10), this section will additionally focus on ratios ranging from 0.1 to 10. To account for variations in PO_4_^3−^ measurements independent of OPs, ten individual PO_4_^3−^ standards at each concentration were prepared in ultrapure water and measured concurrently. The standard deviation of those measurements is represented by the dotted line in Fig. 4, with all SD values < 0.005.

With the PO_4_^3−^ concentration fixed at 2 µM (Fig. 4a and b), the absorbance values for all matrices at both λ_max_ generally fell within the deviation range for the individual PO_4_^3−^ standards in ultrapure water, except in the presence of DTPMP at 710 nm. Notably, starting from a molar ratio of DTPMP:PO_4_^3−^ > 2, a significant increase in absorbance was observed compared to the PO_4_^3−^ standard in ultrapure water. To quantitatively assess the increase in absorbance, the relative differences between the PO_4_^3−^ standards in (i) ultrapure water and (ii) the presence of OPs were calculated (Tables S2–S7). For DTPMP at 710 nm, the absorbance reached 91% at a molar ratio of 3, eventually building up to approximately 200% at a molar ratio of 10.

Similar results were observed for DTPMP when the concentration of PO_4_^3−^ was fixed at 10 µM (Fig. 4c and d). Additionally, for EDTMP, a slight increase in absorbance at 710 nm of approximately 20% was noted starting from a molar ratio of 8. Absorbances at λ_max_ = 880 nm were more robust, with increases in absorbance observed solely for IDMP and DTPMP when the molar ratio reached 6, resulting in values ranging from 10 to 20%.

The increase in absorbance became more pronounced for PO_4_^3−^ concentration fixed at 100 µM, particularly for EDTMP and DTPMP (for quantitative details, see Tables S6 and S7). The highest relative increase in absorbance at λ_max_ = 710 and 880 nm was observed at molar ratio of 10, with values of 81% and 13% for EDTMP, and 202% and 47% for DTPMP, respectively. Moreover, a trend of increasing absorbance at 710 nm in the cases of ATMP and IDMP was also observable. However, the relative increases in the values remained below 20%.

In general, the increase in absorbance due to the presence of OPs was more pronounced at 710 nm compared to 880 nm across all samples tested. This effect intensified with increasing OP concentrations, indicating that the MB reaction involved not only the coloring reagents and PO_4_^3−^ but also the OPs. As a result, varying concentrations of both PO_4_^3−^ and OPs induced spectral changes that disrupted the accuracy of absorbance measurements at 710 or 880 nm. This consequently impairing the linear correlation between PO_4_^3−^ concentration and absorbance, ultimately reflecting as an over-quantification of PO_4_^3−^ concentration across all OP backgrounds. However, implementing straightforward correction factors is challenging in studies involving OP transformation due to the dynamic variations in both PO_4_^3−^ and OP concentrations.

Among the six OPs tested, DTPMP exhibited the most substantial interference in PO_4_^3−^ quantification, likely due to its greater numbers of phosphonic acid groups. Cavaleiro et al. [38] reported that the tartaric acid primarily coordinates with Mo(VI) in a bidentate manner, to form stable five- or six-membered coordination rings. In contrast, organic acids that lack the necessary atom(s) on an α-carbon for the formation of such coordination rings do not appear to interfere with phosphate determination [42]. This also accounts for the lower interference observed for glyphosate and HEDP, as their interaction with Mo(VI) is likely too weak to significantly affect the MB reaction and, consequently, the photometric analysis.

Notably, the significant over-quantification of phosphate is of particular relevance in scenarios involving OP transformations in laboratory experiments, where OP initial concentrations are generally in the high µM to low mM range [11, 27, 43]. However, the interference effects of OPs observed at such high concentrations may not directly translate to environmental conditions, where the phosphate concentrations (0–2 mg/L in rivers [44]) usually are much higher than OP concentrations (0–3 µg/L in rivers and 0–50 µg/L in wastewater effluents [45]). Those typical environmental concentrations align with the experiments containing 2 µM phosphate (~ 0.06 mg/L in P) and OPs-to-phosphate molar ratio of 0.1, corresponding to OP concentration of 0.2 µM (~ 40–110 µg/L for OPs). Under these conditions negligible interference was observed, suggesting that the limitations of the MB method in the presence of OPs are probably irrelevant at environmental concentration levels.

As some OP transformation studies are conducted in more complex matrices containing cations and anions, which potentially influence the formation of the new species identified in this work. To assess the impact of matrix composition, we compared samples prepared in ultrapure water with those in a mixed matrix designed to simulate environmentally relevant conditions. The mixed matrix included earth metal cations (Ca^2+^, Mg^2+^, Fe^2+^, Na^+^, K^+^) and anions (Cl^−^, NO_3_^−^, HCO_3_^−^, SO_4_^2−^). As illustrated in Fig. S6, the matrix did not exert additional effects on phosphate quantification beyond the interferences caused by OPs.

### Case study—measurement of phosphate formation during simulated DTPMP transformation

**Fig. 5:**
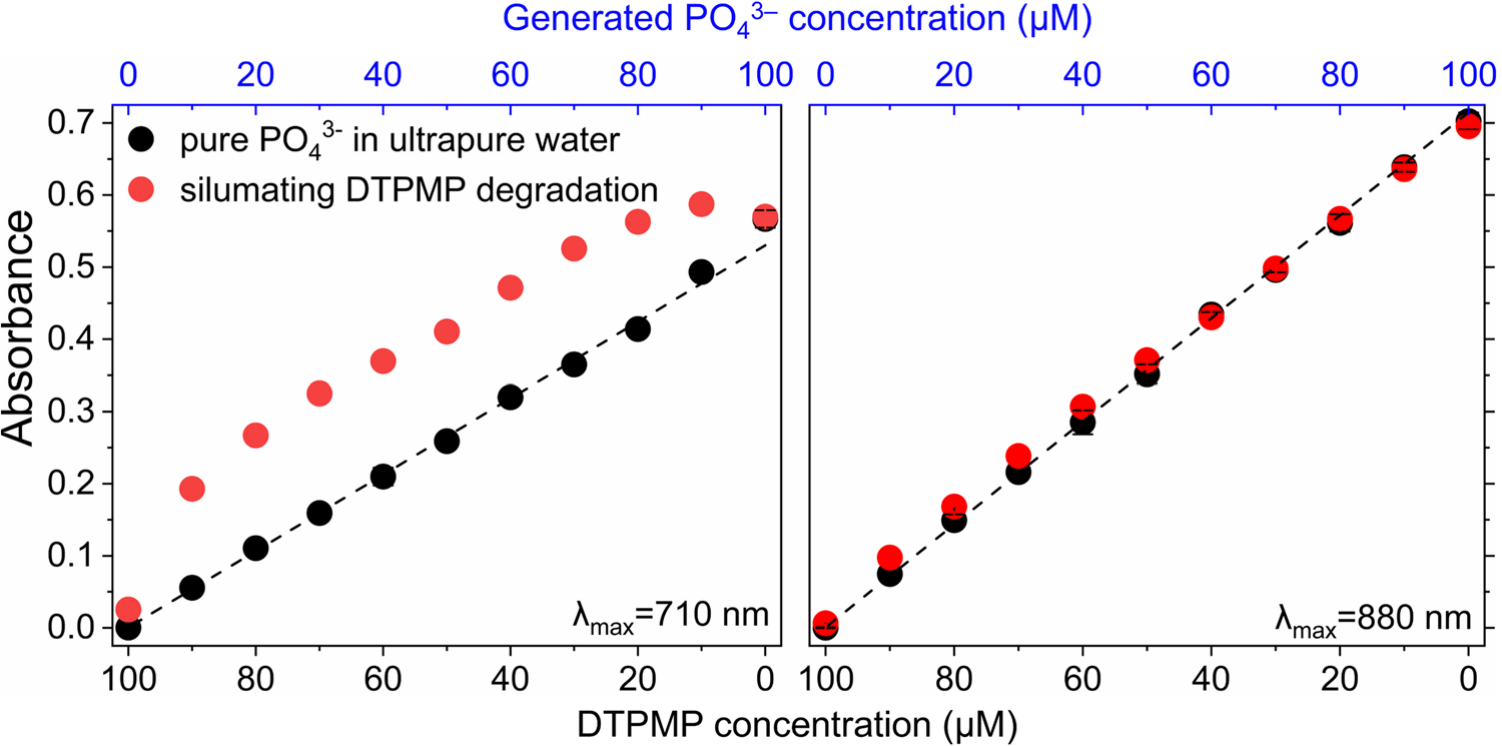
Comparison of absorbance at λ_max_ = 710 nm and 880 nm of PO_4_^3−^ produced during DTPMP degradation with that of pure PO_4_^3−^ standards prepared in ultrapure water at equivalent concentrations. Error bars represent the standard deviation of triplicate experiments. If error bars are not visible, their size is smaller than the marker used to represent the data

In studies investigating OP transformations in laboratory experiments, the formation of PO_4_^3−^ is often used for quantification of reaction progress. However, the coexistence of OPs and PO_4_^3−^ throughout the transformation process impairs PO_4_^3−^ quantification, as demonstrated by the findings in this study. To assess the potential over-quantification of PO_4_^3−^ concentration by the MB method, a typical laboratory DTPMP degradation experiment was simulated with an initial concentration of 1 mM and under the assumption that equimolar concentration of PO_4_^3−^ is formed as DTPMP is degraded. Eleven samples with differing PO_4_^3−^ and DTPMP concentrations mimicking different stages of the degradation process were prepared. The absorbance of the simulated degradation samples was compared with that of PO_4_^3−^ standards in ultrapure water at corresponding concentrations (Fig. 5).

Quantification using the MB method revealed substantially higher absorbances at 710 nm for the simulated degradation samples compared to PO_4_^3−^ standards of equivalent concentration prepared in ultrapure water. For instance, the absorbance corresponding to the generation of 10 µM PO_4_^3−^ in DTPMP degradation samples was approximately equivalent to that of 40 µM pure PO_4_^3−^ standards. The relative over-quantification of PO_4_^3−^ concentration at 810 nm was significantly lower than that observed at 710 nm. Specifically, the relative increase in absorbance for the DTPMP degradation samples containing 40 µM PO_4_^3−^, when compared to equivalent pure PO_4_^3−^ standards, was only 8%. However, this deviation cannot be ignored, particularly during the early stages of DTPMP degradation when the molar ratio is relatively high.

## Conclusion

This study highlights significant limitations of the MB method for PO_4_^3−^ quantification in the presence of OP chelating agents. The results showed substantial changes in the absorption spectra, characterized by the appearance of a new absorption maximum at 760 nm. The original λ_max_ at 710 nm and 880 nm were shifted to longer wavelengths, accompanied by increased absorbance at these two λ_max_. These phenomena are attributed to the formation of new colored species involving PO_4_^3−^, molybdate, and OPs, which disrupted the linear relationship between absorbance values and PO_4_^3−^ concentration, resulting in quantification inaccuracies.

Single-factor experiments demonstrated concentration-dependent interference, with higher concentrations of both substances exacerbating quantification inaccuracies. Notably, significant over-quantification of PO_4_^3−^ concentration was observed in the presence of larger OPs, such as EDTMP and DTPMP, particularly when the OPs-to-PO_4_^3−^ molar ratio exceeded 1. The relative increases in absorbance were less pronounced at λ_max_ = 880 nm, suggesting 880 nm is more reliable for PO_4_^3−^ quantification. Among the OPs tested, DTPMP exhibited the strongest impact on absorbance values at both λ_max_, while glyphosate showed minimal effects. A case study simulating DTPMP degradation revealed over-quantification of PO_4_^3−^ by up to 350%, especially during early transformation stages, highlighting the method’s limitations in such contexts.

In view of these challenges, although the MB method remains a valuable tool for rapid PO_4_^3−^ quantification, its limitations require careful consideration of the specific OPs involved and their concentrations, particularly in OP transformation research at high concentrations. However, our results indicate negligible interference at low OP and PO_4_^3−^ concentrations, suggesting that the limitations of the MB method are probably irrelevant at environmentally relevant concentrations. To address these limitations in more complex matrices, future research should prioritize the development and application of complementary analytical techniques, such as chromatographic separation, post-column reactions prior to spectrometry, or the utilization of IC-ICP-MS, to improve the accuracy and reliability of PO_4_^3−^ quantification.

## Supplementary Information

Below is the link to the electronic supplementary material.

ESM 1 (DOCX 6.92 MB)

## Data Availability

The datasets generated during and/or analyzed during the current study are available from the corresponding author on reasonable request.
