# Supplementary material for: Limitations of the molybdenum blue method for phosphate quantification in the presence of organophosphonates
Source: Anal Bioanal Chem. 2025 Mar 31;417(14):3103–11. doi: 10.1007/s00216-025-05850-y (PMC12103472; doi:10.1007/s00216-025-05850-y)
Supplement: Supplementary file 1 — (DOCX 6.92 MB) [file 216_2025_5850_MOESM1_ESM.docx]

Supplementary Information (SI) to:

**Limitations of the molybdenum blue method for**

**phosphate quantification in the presence of organophosphonates**

*Ruoning Guo^a^, Anna M. Röhnelt^a^, Philipp R. Martin^a,1^, Stefan B. Haderlein^a*^*

*a. Geo- and Environmental Research Center, Department of Geosciences, Eberhard Karls Universität Tübingen, 72076 Tübingen, Germany*

*1. Present address: Division of Environmental Geosciences, Centre for Microbiology and Environmental Systems Science, University of Vienna, 1090 Wien, Austria*

** Corresponding author:* [*stefan.haderlein@uni-tuebingen.de*](mailto:stefan.haderlein@uni-tuebingen.de)

Total number of pages (including cover): 21

Figures: 6

Tables: 9

**Table S1.** Reaction conditions of the MB method regulated by different standardized protocols.

|  | Water quality - Determination of phosphorus - Ammonium molybdate spectrometric method (ISO 6878:2004) | Method 365.3: Phosphorous, All Forms (Colorimetric, Ascorbic Acid, Two Reagent) | Standard methods for the examination of water and wastewater (the MB_APHA_ method) | Our method (the MB_Tü_ method) |
| --- | --- | --- | --- | --- |
| reagent A | 0.842 mM ammonium molybdate | 0.453 mM ammonium molybdate | 0.777 mM ammonium molybdate | 1.683 mM ammonium molybdate |
| reagent B | 0.042 mM antimony potassium tartrate | 0.021 mM antimony potassium tartrate | 0.033 mM antimony potassium tartrate | 0.090 mM antimony potassium tartrate |
| reagent C | 0.216 M H_2_SO_4_ | 0.096 M H_2_SO_4_ | 0.200 M H_2_SO_4_ | 0.368 M H_2_SO_4_ |
| reagent D | 0.011 M  ascorbic acid | 0.012 M  ascorbic acid | 0.005 M  ascorbic acid | 0.011 M  ascorbic acid |
| reaction time | 10‒30 min | 5 min | 10‒30 min | 10‒30 min |
| Measured at which wavelength | 880 nm or 700 nm | 650 nm | 880 nm | 710 nm and 880 nm |
| Reference | (1) | (2) | (3) | (4) |

**Nuclear magnetic resonance spectroscopy (NMR)**

To ensure the purity of the acquired EDTMP and DTPMP, ^31^P-{^1^H}-NMR was conducted at NMR department of Chemistry Department, University of Tübingen. ^31^P-NMR spectroscopy is an effective analytical technique for assessing the purity of phosphonates due to high sensitivity and resolution by exploiting 100% natural abundance and the broad chemical shift range of ^31^P (5).

10 mg of DTPMP resp. EDTMP and 600 μL of deuterated water (D_2_O, 99.9 atom% D, obtained by Sigma-Aldrich, Steinheim, Germany) was vortexed for 5 s, respectively. Subsequently, 600 μL of the solution was transferred to an NMR glass tube. NMR measurements were conducted using a Bruker AMX 600 MHz NMR spectrometer (Bruker, Billerica, MA, USA), operating at 242.94 MHz for phosphorous observation with a zgpg30 pulse program, and at 600.13 MHz for hydrogen observation employing the zg30 pulse program in the case of EDTMP analysis. The acquisition parameters used for this experiment with 1D sequence with power-gated decoupling and a 30 ° flip angle were as follows for ^31^P (^1^H): number of scans set to 64 (32), spectral width at 96153.84 Hz (12019.23 Hz), offset (O1) at -12146.85 Hz (3705.80 Hz), acquisition time of 0.34 s (2.73 s), and relaxation delay (d1) of 2.00 s (1.00 s). The resulting spectrum was quantitatively analyzed using Bruker Top Spin version 4.1.4 software.

The ^1^H-NMR-spectrum and ^31^P-{^1^H}-NMR-spectrum of EDTMP were shown in Fig. S1 and S2, respectively. The signal integrals are normalized to a total of 100. Impurities are indicated with an asterisk. The ^1^H-NMR-spectrum reveals signals attributed to EDTMP in a ratio of 4:8. The singlet at δ (ppm) 3.88 corresponds to the protons of the ethylenediamine moiety in the middle and the duplet at δ (ppm) 3.52 represents the protons of the four phosphonate groups attached to the amine moiety. The impurities of the analysed EDTMP amount for only 2.52%, proving that the EDTMP used in the experiments has a high purity of 97.48%. In Fig. S2, one signal corresponding to EDTMP is observed, representing all chemically equivalent phosphonate groups at δ (ppm) 8.76. The impurities containing phosphorous in the analysed EDTMP amount to only 3.40%, confirming that EDTMP used in the experiments is of high purity regarding P with 96.60%.

In the ^31^P-{^1^H}-NMR-spectrum of DTPMP (Fig. S3), two main signals in a ratio of 1:4 can be seen, which represent the phosphonate-group in the middle of DTPMP (δ (ppm): 12.94) and the four phosphonate groups of DTPMP attached to the outer amine moieties (δ (ppm): 9.23). Impurities are marked with an asterisk. The sum of all signal-integrals is normalized to 100. Impurities of the analysed DTPMP contribute 1.37%. The purity of DTPMP used in the experiments regarding P is therefore >98.6%.


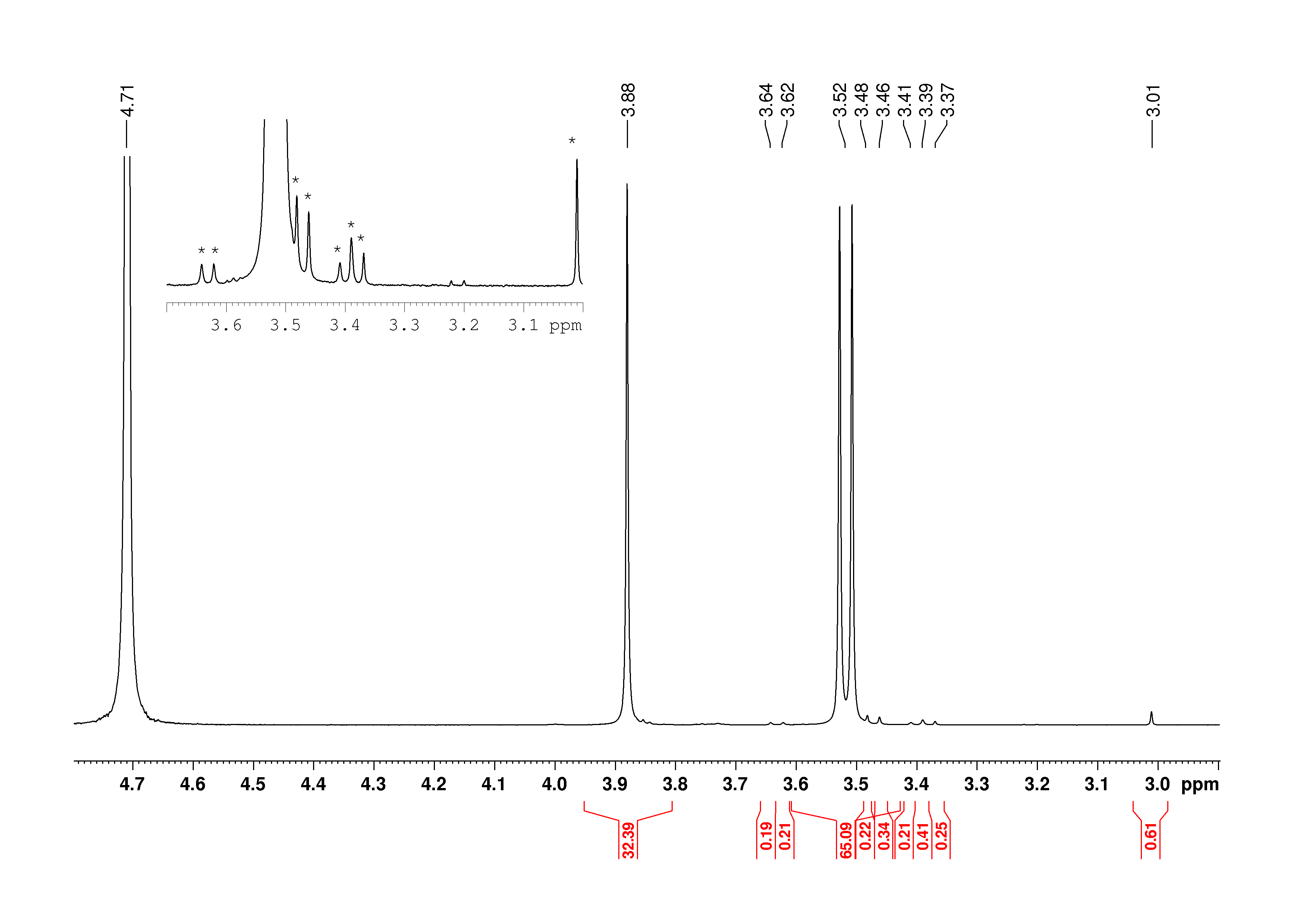


***Fig. S1*** *^1^H-NMR-**spectrum of EDTMP in D_2_O measured as stated in section NMR. δ (ppm): 9.23, 12.94. Impurities are marked with an asterisk. The sum of integrals is normalized to 100*


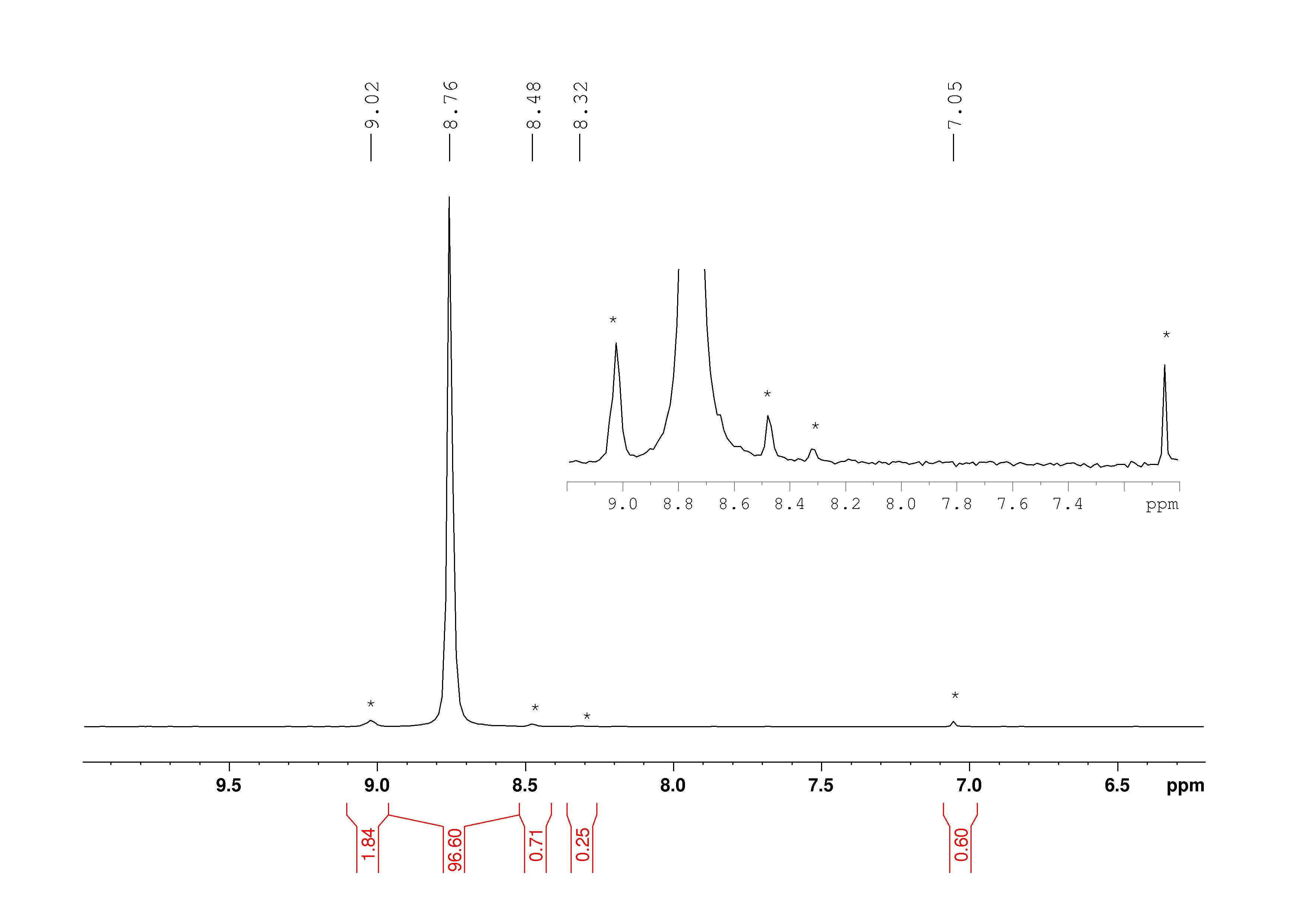


***Fig. S2*** *^31^P-{^1^H}-NMR-spectrum of EDTMP in D_2_O measured as stated in section NMR. δ (ppm): 8.76. Impurities are marked with an asterisk. The sum of integrals is normalized to 100*

**

***Fig. S3*** *^31^P-{^1^H}-NMR-spectrum of DTPMP in D_2_O measured as stated in section NMR. δ (ppm): 9.23, 12.94. Impurities are marked with an asterisk. The sum of integrals is normalized to 100*

**Ion chromatography coupled to inductively coupled plasma mass spectrometry (IC-ICP-MS)**

For the determination of phosphate impurities, a prep*FAST* IC system from Elemental Scientific (Omaha, NE, USA) hyphenated to an iCAP TQ inductively coupled plasma-mass spectrometer (ICP-MS) from Thermo Fisher Scientific (Bremen, Germany) was used.

All species were separated by an anion exchange column CF-Cr-01 (Elemental Scientific) with a flow rate of 1000 µL/min. Eluents were prepared with nitric acid (HNO_3_, 65%) from Thermo Fisher Scientific, which was purified with a DST-1000 acid purification system from Savillex (Eden Prairie, MN, USA), ammonia solution (25-27%, for trace analysis) from VWR International LLC (Radnor, PA, USA), diethylenetriaminepentaacetic acid (DTPA) from Honeywell/Fluka (Charlotte, NC, USA) and doubly distilled water from a Aquatron A4000D system from Barloworld Scientific (Nemours, France). Eluent A consisted of 300 µg/L DTPA at pH 9.2 and eluent B of 150 mM ammonium nitrate with 300 µg/L DTPA at pH 9.2. Gradient timings were 8.5% eluent B for 0-15 s, 20.0% eluent B for 15-65 s, 40.0% eluent B for 65-110 s, 70.0% eluent B for 110-150 s and 93.0% eluent B for 150-205 s. The injection volume was set to 50 µL. In in 2% nitric acid (1 µg/L) at a flow rate of 100 µL/min served as a post-column internal standard and was purchased from Sigma-Aldrich (St. Louis, MO, USA). Phosphorus (^31^P^16^O^+^) and indium (^115^In^+^) were detected in triple-quadruple oxygen mode with dwell times of 100 ms. Phosphate IC-standard solution (1000 mg/L PO_4_^3-^ in H_2_O) from Merck (Darmstadt, Germany) was used for the external calibration of phosphate. LOD and LOQ were determined by the 3σ and 10σ criteria.

Injections of 30 ppb P of each species were analyzed to determine phosphate impurities. The chemical standards utilized for IC-ICP-MS were consistent with those outlined in the Materials and Methods section of the manuscript. In the IC-ICP-MS chromatogram (Fig. S4), minor phosphate peaks were detected in the OP standards. Quantitative integration of these peaks indicated that the phosphate concentrations were negligible, falling below the limit of detection (LOD, 0.0041 µM) or limit of quantification (LOQ, 0.0138 µM). Therefore, phosphate impurity levels are given as maximum value based on the LOD or LOQ, ranging from 0.2 to 2.7 mol-%. Comprehensive analytical data for each analyte are provided in Table S1.





***Fig. S4*** *Phosphorus-selective IC-ICP-MS chromatograms of individually injected OPs.*

**Table S2.** Quantification of phosphate impurities in OP standards quantified by IC-ICP-MS, mol-% are given as a maximum value based on the LOD or LOQ.

|  | c / µM analyte | c (phosphate) / µM | LOD/LOQ phosphate / µM | Impurity of phosphate molar (max.) |
| --- | --- | --- | --- | --- |
| Glyphosate | 1.003 | <LOD | 0.0041 | <0.4% |
| IDMP | 0.503 | <LOD | 0.0138 | <2.7% |
| ATMP | 0.336 | <LOQ | 0.0041 | <1.2% |
| EDTMP | 0.251 | <LOD | 0.0041 | <1.6% |
| DTPMP | 0.201 | <LOD | 0.0041 | <2.0% |

*
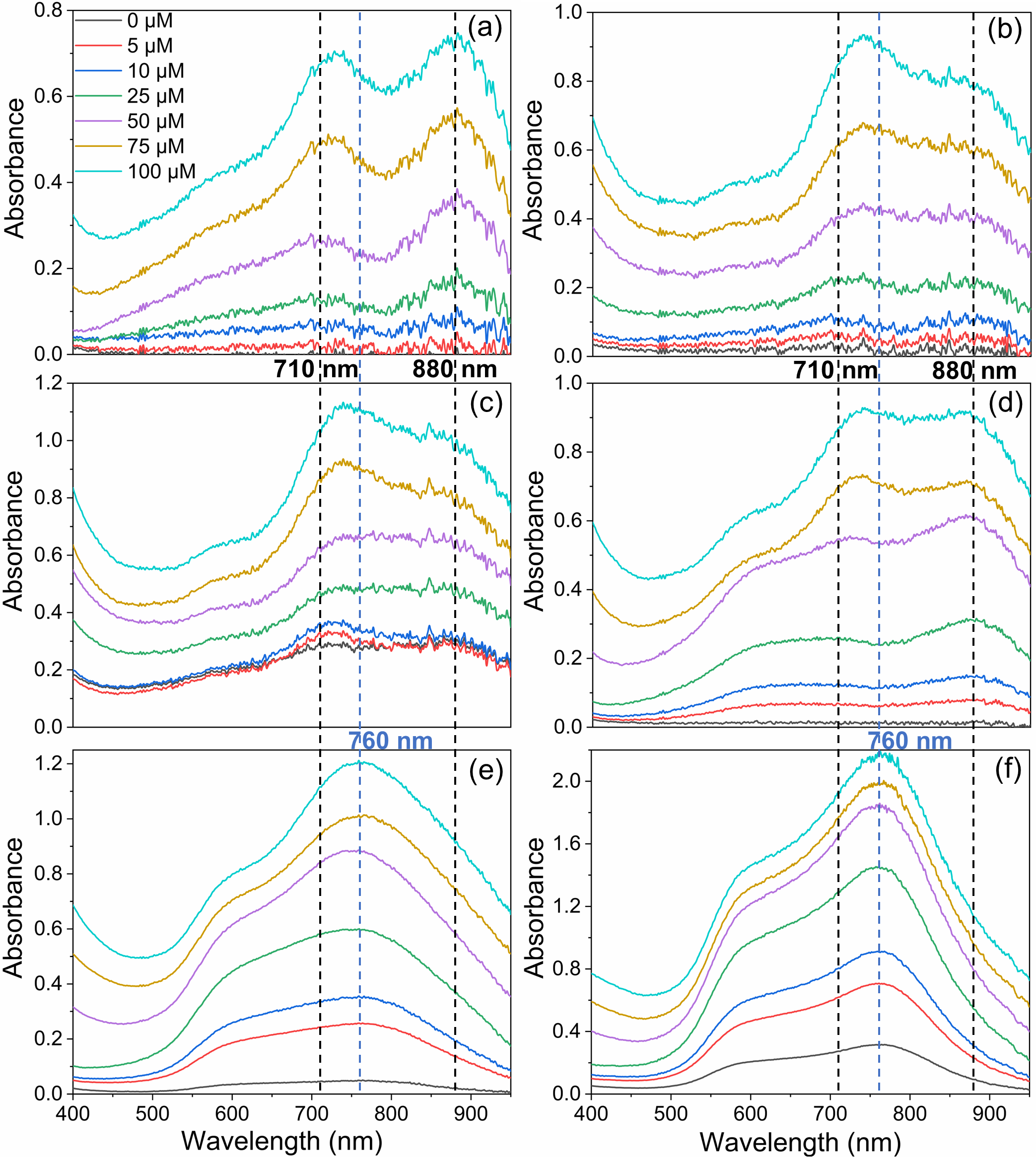
*

***Fig. S5*** *Absorption spectra of mix-standards containing PO_4_^3‒^ at varying concentrations and 1 mM of (a) glyphosate, (b) HEDP, (c) IDMP, (d) ATMP, (e) EDTMP, and (f) DTPMP*

**Table S3.** Relative increase in absorbance* for mix-standards containing varying concentration of PO_4_^3‒^ and 1 mM of individual OPs, compared to the absorbance of PO_4_^3‒^ standards in ultrapure water.

|  | PO_4_^3‒^ concentration (µM) | glyphosate | HEDP | IDMP | ATMP | EDTMP | DTPMP |
| --- | --- | --- | --- | --- | --- | --- | --- |
| λ_max_=710 nm (%) | 5 | -33.78 | 137.36 | 839.92 | 109.50 | 734.50 | 1979.15 |
|  | 10 | 15.86 | 113.40 | 520.49 | 117.58 | 558.63 | 1473.61 |
|  | 25 | -3.11 | 74.81 | 218.93 | 77.62 | 323.84 | 826.09 |
|  | 50 | -0.96 | 57.11 | 120.22 | 75.12 | 202.85 | 500.81 |
|  | 75 | 12.52 | 52.23 | 97.76 | 58.48 | 129.98 | 332.22 |
|  | 100 | 8.42 | 35.88 | 56.43 | 32.16 | 79.57 | 214.92 |
|  |  |  |  |  |  |  |  |
| λ_max_=880 nm (%) | 5 | 17.96 | 89.20 | 726.30 | 76.84 | 318.21 | 631.00 |
|  | 10 | 64.43 | 100.76 | 427.64 | 122.11 | 254.90 | 480.76 |
|  | 25 | 36.07 | 56.55 | 197.16 | 100.26 | 160.18 | 282.65 |
|  | 50 | 35.80 | 48.11 | 111.46 | 91.96 | 103.88 | 181.63 |
|  | 75 | 36.00 | 46.69 | 81.42 | 59.98 | 74.54 | 127.86 |
|  | 100 | 21.64 | 27.97 | 49.63 | 37.64 | 45.60 | 81.08 |

** Relative increase in absorbance were calculated in percentages (%): = (absorbance of solutions containing 1 mM OPs/absorbance of PO_4_^3‒^ standard in ultrapure water-1) × 100%*

**Table S4.** Relative increase in absorbance at λ_max_=710 nm for 2 µM PO_4_^3‒^ standards containing varying concentrations of OPs, compared to the absorbance of PO_4_^3‒^ standards in ultrapure water.

|  | Relative increase in absorbance at 710 nm* (%) | | | | | |
| --- | --- | --- | --- | --- | --- | --- |
| OPs to PO_4_^3‒^ molar ratio | glyphosate | HEDP | IDMP | ATMP | EDTMP | DTPMP |
| 0 | 16.62 | -13.84 | 18.36 | -5.13 | 17.93 | 19.5 |
| 0.1 | 26.46 | 17.23 | 7.31 | -11.23 | -6.27 | 31.42 |
| 0.2 | 37.77 | 19.5 | 20.37 | -19.06 | -23.67 | 53.18 |
| 0.3 | 20.71 | 15.75 | 5.05 | -18.8 | -12.36 | 8.53 |
| 0.4 | 28.55 | -5.4 | 10.53 | -8.36 | -3.13 | 15.14 |
| 0.5 | 15.14 | -1.65 | 1.83 | -6.61 | 17.23 | 42.12 |
| 0.6 | 36.03 | 2.7 | 1.83 | -0.78 | -10.97 | 24.72 |
| 0.7 | 20.1 | -13.23 | 12.88 | -25.76 | -2.52 | 2.7 |
| 0.8 | 23.59 | 4.44 | 10.53 | -16.19 | -26.89 | 34.64 |
| 0.9 | 11.14 | -5.74 | -0.17 | -32.38 | -20.54 | 16.62 |
| 1 | 53.79 | 4.7 | 16.36 | 7.05 | 4.44 | 26.46 |
| 2 | 21.58 | 17.49 | 0.09 | -11.84 | -11.84 | 33.77 |
| 3 | 20.97 | -12.71 | -12.97 | -17.32 | 4.7 | 90.86 |
| 4 | 2.7 | 1.22 | 45.95 | -7.75 | 8.18 | 57.53 |
| 5 | -6.61 | 28.55 | 7.92 | -16.45 | 5.05 | 64.75 |
| 6 | 7.05 | 14.27 | 36.38 | 0.7 | -6.88 | 90.6 |
| 7 | 20.71 | 19.84 | 28.81 | -7.48 | 10.27 | 129.5 |
| 8 | 12.88 | 19.5 | 9.92 | 14.27 | -8.62 | 134.38 |
| 9 | 7.31 | 19.23 | 25.33 | -19.67 | -9.49 | 177.63 |
| 10 | 37.51 | 16.88 | 10.27 | -10.97 | -10.36 | 202.26 |

** Data = (AVG. absorbance of triplicate samples/AVG. absorbance of PO4 standards -1) ×100%*

**Table S5.** Relative increase in absorbance at λ_max_=880 nm for 2 µM PO_4_^3‒^ standards containing varying concentrations of OPs, compared to the absorbance of PO_4_^3‒^ standards in ultrapure water.

|  | Relative increase in absorbance at 880 nm (%) | | | | | |
| --- | --- | --- | --- | --- | --- | --- |
| OPs to PO_4_^3‒^ molar ratio | glyphosate | HEDP | IDMP | ATMP | EDTMP | DTPMP |
| 0 | 15.62 | -12.18 | 17.14 | -1.85 | 12.51 | 14.49 |
| 0.1 | 25.55 | 13.83 | 6.35 | -2.91 | -7.54 | 23.76 |
| 0.2 | 43.15 | 20.25 | 23.76 | -14.43 | -24.09 | 37.19 |
| 0.3 | 21.97 | 16.28 | 3.9 | -12.44 | -13.1 | -7.54 |
| 0.4 | 20.91 | -3.57 | 10.52 | -0.8.67 | -6.22 | -1.85 |
| 0.5 | 15.16 | 1.26 | 3.71 | -6.02 | 9.66 | 24.42 |
| 0.6 | 33.88 | 4.37 | 2.58 | 1.46 | -14.43 | 5.03 |
| 0.7 | 17.14 | -8.87 | 13.83 | -21.71 | -2.05 | -15.75 |
| 0.8 | 21.77 | 6.09 | 12.31 | -13.3 | -26.34 | 12.31 |
| 0.9 | 8.07 | -4.24 | 2.58 | -30.97 | -21.05 | -0.93 |
| 1 | 46.72 | 5.43 | 16.68 | 6.09 | 0.4 | 1.26 |
| 2 | 19.99 | 16.28 | 1.26 | -7.35 | -13.1 | -10.85 |
| 3 | 21.58 | -10.66 | -9.53 | -13.96 | 1.72 | 22.24 |
| 4 | 4.1 | 1.92 | 44.94 | -4.5 | 2.78 | -21.44 |
| 5 | -4.7 | 27.27 | 10.52 | -9.53 | -0.73 | -29.19 |
| 6 | 10.52 | 12.05 | 34.55 | 0.6 | -9.53 | -15.49 |
| 7 | 20.65 | 16.68 | 29.05 | -9.13 | 5.69 | 4.37 |
| 8 | 6.35 | 14.96 | 10.32 | 14.69 | -6.88 | -3.18 |
| 9 | 8.54 | 16.48 | 26.6 | -18.13 | -13.77 | 24.22 |
| 10 | 33.49 | 13.83 | 13.37 | -9.99 | -14.82 | 37.19 |

**Table S6.** Relative increase in absorbance at λ_max_=710 nm for 10 µM PO_4_^3‒^ standards containing varying concentrations of OPs, compared to the absorbance of PO_4_^3‒^ standards in ultrapure water.

|  | Relative increase in absorbance at 710 nm (%) | | | | | |
| --- | --- | --- | --- | --- | --- | --- |
| OPs to PO_4_^3‒^ molar ratio | glyphosate | HEDP | IDMP | ATMP | EDTMP | DTPMP |
| 0 | -7.07 | -9.8 | -7.31 | -4.16 | -6.58 | -16.77 |
| 0.1 | -4.47 | -9.13 | -3.31 | -8.35 | -7.57 | -4.16 |
| 0.2 | -5.26 | -10.53 | -4.58 | -3.26 | -7.86 | -2.16 |
| 0.3 | -9.62 | -10.95 | 0.87 | -6.04 | -6.16 | 0.56 |
| 0.4 | -9.75 | -13.2 | 1.78 | -6.84 | -8.4 | 5.29 |
| 0.5 | -10.17 | -6.11 | -8.71 | -7.67 | -7.86 | 10.87 |
| 0.6 | -3.13 | -9.13 | -1.8 | -7.57 | -7.67 | 17.17 |
| 0.7 | -7.44 | -10.89 | -4.58 | -8.47 | -5.62 | 16.2 |
| 0.8 | -3.49 | -1.67 | -7.2 | -5.49 | -5.67 | 21.79 |
| 0.9 | -6.66 | -11.31 | -2.84 | -2.35 | -1.67 | 23.84 |
| 1 | -5.67 | -9.44 | -7.07 | -4.22 | -6.89 | 27.35 |
| 2 | -4.53 | -10.35 | -1.25 | -2.35 | -5.07 | 63.67 |
| 3 | -2.58 | -3.44 | -1.13 | 1.05 | -3.86 | 87.07 |
| 4 | -5.38 | -3.26 | 0.15 | 2.87 | -0.65 | 114.58 |
| 5 | -1.93 | -5.02 | 2.75 | 1.89 | -0.22 | 135.99 |
| 6 | -3.2 | -3.13 | 13.11 | 3.16 | 7.35 | 158.9 |
| 7 | -8.53 | -0.71 | 10.69 | 4.02 | 8.2 | 184.91 |
| 8 | -9.93 | -1.07 | 13.78 | 5.24 | 15.29 | 199.87 |
| 9 | -6.84 | 5.24 | 16.44 | 5.84 | 16.02 | 220.73 |
| 10 | -11.49 | -0.95 | 19.29 | 7.78 | 20.15 | 239.39 |

**Table S7.** Relative increase in absorbance at λ_max_=880 nm for 10 µM PO_4_^3‒^ standards containing varying concentrations of OPs, compared to the absorbance of PO_4_^3‒^ standards in ultrapure water.

|  | Relative increase in absorbance at 880 nm (%) | | | | | |
| --- | --- | --- | --- | --- | --- | --- |
| OPs to PO_4_^3‒^ molar ratio | glyphosate | HEDP | IDMP | ATMP | EDTMP | DTPMP |
| 0 | -6.89 | -9.9 | -7.65 | -5.58 | -4.94 | -13.1 |
| 0.1 | -4.67 | -8.32 | -4.27 | -9.36 | -6.35 | -6.43 |
| 0.2 | -5.08 | -10.08 | -4.27 | -5.54 | -6.48 | -8.65 |
| 0.3 | -9.19 | -10.81 | -0.27 | -7.83 | -5.54 | -9.77 |
| 0.4 | -9.4 | -13.01 | -0.85 | -8.42 | -7.29 | -9.09 |
| 0.5 | -9.63 | -6.93 | -9 | -9.5 | -6.79 | -6.89 |
| 0.6 | -3.42 | -10.17 | -3.78 | -9.36 | -6.84 | -4.46 |
| 0.7 | -7.24 | -12.16 | -5.35 | -10.48 | -5.4 | -8.24 |
| 0.8 | -4.67 | -3.24 | -7.51 | -8.01 | -5.48 | -5.98 |
| 0.9 | -6.48 | -12.24 | -3.69 | -5.48 | -4.59 | -7.38 |
| 1 | -5.81 | -10.13 | -7.24 | -6.25 | -6.43 | -7.11 |
| 2 | -4.59 | -12.64 | -2.11 | -6.35 | -6.79 | -0.31 |
| 3 | -3.05 | -6.93 | -2.38 | -3.69 | -6.84 | 0.32 |
| 4 | -5.48 | -7.47 | -0.99 | -2.47 | -6.16 | 3.2 |
| 5 | -2.2 | -9.73 | 1.13 | -3.92 | -7.7 | 5.73 |
| 6 | -3.38 | -8.96 | 9.73 | -3.42 | -3.15 | 8.92 |
| 7 | -8.32 | -6.75 | 8.7 | -2.65 | -4.27 | 15.18 |
| 8 | -9.5 | -7.51 | 10.73 | -1.43 | -0.45 | 12.43 |
| 9 | -6.7 | -2.61 | 12.89 | -1.7 | -1.97 | 16.8 |
| 10 | -10.81 | -8.65 | 15.32 | -0.41 | -1.35 | 17.43 |

**Table S8.** Relative increase in absorbance at λ_max_=710 nm for 100 µM PO_4_^3‒^ standards containing varying concentrations of OPs, compared to the absorbance of PO_4_^3‒^ standards in ultrapure water.

|  | Relative increase in absorbance at 710 nm (%) | | | | | |
| --- | --- | --- | --- | --- | --- | --- |
| OPs to PO_4_^3‒^ molar ratio | glyphosate | HEDP | IDMP | ATMP | EDTMP | DTPMP |
| 0 | 6.03 | 1.91 | -2.56 | 2.22 | -2.02 | -4.38 |
| 0.1 | 7.91 | -3.32 | -2.53 | -0.66 | 2.04 | 6.72 |
| 0.2 | 7.83 | -3.65 | -1.63 | -0.14 | 4.35 | 12.66 |
| 0.3 | 6.37 | 2.74 | -2.28 | 3.5 | 3.36 | 17.75 |
| 0.4 | 5.7 | -4.88 | -1.71 | -0.74 | 3.34 | 22.92 |
| 0.5 | 6.82 | -4.4 | 0.61 | 2.7 | 5.21 | 28.03 |
| 0.6 | 6.32 | -4.48 | 1.37 | 4.62 | 3.85 | 31.27 |
| 0.7 | 7.55 | -2.53 | 1.35 | 3.03 | 1.21 | 32.91 |
| 0.8 | 7.38 | -0.03 | 2.2 | 6.94 | 4.31 | 37.1 |
| 0.9 | 7.76 | -6.18 | 0.83 | 5.46 | 4.68 | 36.64 |
| 1 | 6.82 | -6.23 | 4.23 | 6.88 | 8.1 | 45.59 |
| 2 | 6.94 | 5.28 | 4.05 | 2.11 | 16.38 | 64.65 |
| 3 | 6.22 | -0.86 | 10.87 | 2.51 | 23.13 | 90.79 |
| 4 | 5.87 | 1.8 | 5.49 | 4.21 | 31.34 | 109.65 |
| 5 | 5.52 | -4.9 | 9.78 | 2.69 | 37.54 | 132.36 |
| 6 | 7.41 | 1.75 | 10.23 | 4.54 | 44.15 | 150.37 |
| 7 | 0.21 | -4.88 | 9.21 | 7 | 55.54 | 164.84 |
| 8 | 3.72 | -4.92 | 11.67 | 6.46 | 61.83 | 195.64 |
| 9 | 0.52 | -3.58 | 13.26 | 8.43 | 72.93 | 203.59 |
| 10 | -3.62 | 4.14 | 14.34 | 8.42 | 81.03 | 202.2 |

**Table S9.** Relative increase in absorbance at λ_max_=880 nm for 100 µM PO_4_^3‒^ standards containing varying concentrations of OPs, compared to the absorbance of PO_4_^3‒^ standards in ultrapure water.

|  | Relative increase in absorbance at 880 nm (%) | | | | | |
| --- | --- | --- | --- | --- | --- | --- |
| OPs to PO_4_^3‒^ molar ratio | glyphosate | HEDP | IDMP | ATMP | EDTMP | DTPMP |
| 0 | 1.42 | 2.59 | -2.52 | -3.98 | 0.06 | -0.86 |
| 0.1 | 0.56 | 1.15 | -6.34 | -3.94 | -1.11 | -1.46 |
| 0.2 | 0.87 | 0.99 | -7.04 | -4.18 | -1.24 | -1.38 |
| 0.3 | 0.5 | 1.12 | -5.12 | -4.61 | -2.11 | -1.29 |
| 0.4 | 0.36 | 0.15 | -8.82 | -4.03 | -2.4 | -0.8 |
| 0.5 | 0.43 | -0.28 | -7.76 | -4.16 | -2.27 | -0.22 |
| 0.6 | 1.31 | -0.25 | -8.37 | -4.16 | -2.96 | 0.26 |
| 0.7 | 0.85 | -1.04 | -6.97 | -3.89 | -2.47 | 0.13 |
| 0.8 | 0.68 | -0.43 | -6.17 | -4.61 | -3.41 | 0.92 |
| 0.9 | 1.77 | -1.69 | -9.26 | -4.39 | -2.96 | 0.18 |
| 1 | 1.35 | -1.39 | -9.5 | -3.54 | -2.13 | 3.92 |
| 2 | 0.73 | 0.47 | -4.67 | -4.39 | 0.51 | 3.69 |
| 3 | 0.84 | 1.7 | -7.17 | -2.01 | 1.49 | 10.83 |
| 4 | 0.3 | 0.06 | -6 | -3.34 | 4.17 | 12.17 |
| 5 | 0.47 | 0.23 | -7.61 | -0.42 | 5.2 | 20.16 |
| 6 | 1.56 | 0.13 | -6.07 | 0.3 | 5.99 | 24.29 |
| 7 | -0.88 | 5.06 | -9.81 | -0.39 | 8.35 | 28.13 |
| 8 | 1.02 | -1.79 | -9.51 | 6.7 | 9.43 | 41.24 |
| 9 | -1.08 | -2.42 | -9.46 | 2.17 | 11.88 | 44.02 |
| 10 | -2.65 | -4.34 | -5.57 | 3.61 | 13.37 | 47.32 |

**Effects of matrix composition**

In order to investigate the effects of matrix, we conducted additional experiments comparing samples in ultrapure water with those in a mixed matrix designed to simulate environmentally relevant conditions. We focused on DTPMP-phosphate mixed samples, as they demonstrated the most significant impact in our study. To systematically evaluate the effect of matrix, we selected five DTPMP-to-phosphate molar ratios under three fixed phosphate concentrations, consistent with the conditions presented in Fig. 4 of the manuscript. The mixed matrix included the following components: 1 mM CaCl_2_, 0.5 mM KNO_3_, 0.5 mM NaHCO_3_, 0.5 mM MgSO_4_·7H_2_O, and 3.6 µM FeSO_4_·7H_2_O. These concentrations were selected based on prior research investigating the influence of bivalent metal ions on the photolysis of PPs (8). All chemicals used were of high purity (≥98–99.5%) and obtained from reputable suppliers (Merck, Sigma Aldrich, and Carl Roth).

Our results, as shown in Fig. S6, demonstrate that the presence of the mixed matrix did not significantly influence phosphate quantification. Specifically, for samples with 2 µM phosphate, the observed differences could be attributed to the standard deviation associated with quantifying low phosphate concentrations. For samples with 10 µM phosphate, a slight increase in absorbance (within 30%) was noted at two λ_max_ for samples in matrix. However, the spectral profiles remained unchanged compared to those of DTPMP-phosphate mixed samples in ultrapure water, confirming that the interference of DTPMP was not affected by the matrix. Moreover, for cases with 100 µM phosphate, absorbances in ultrapure water and the mixed matrix were identical.

**
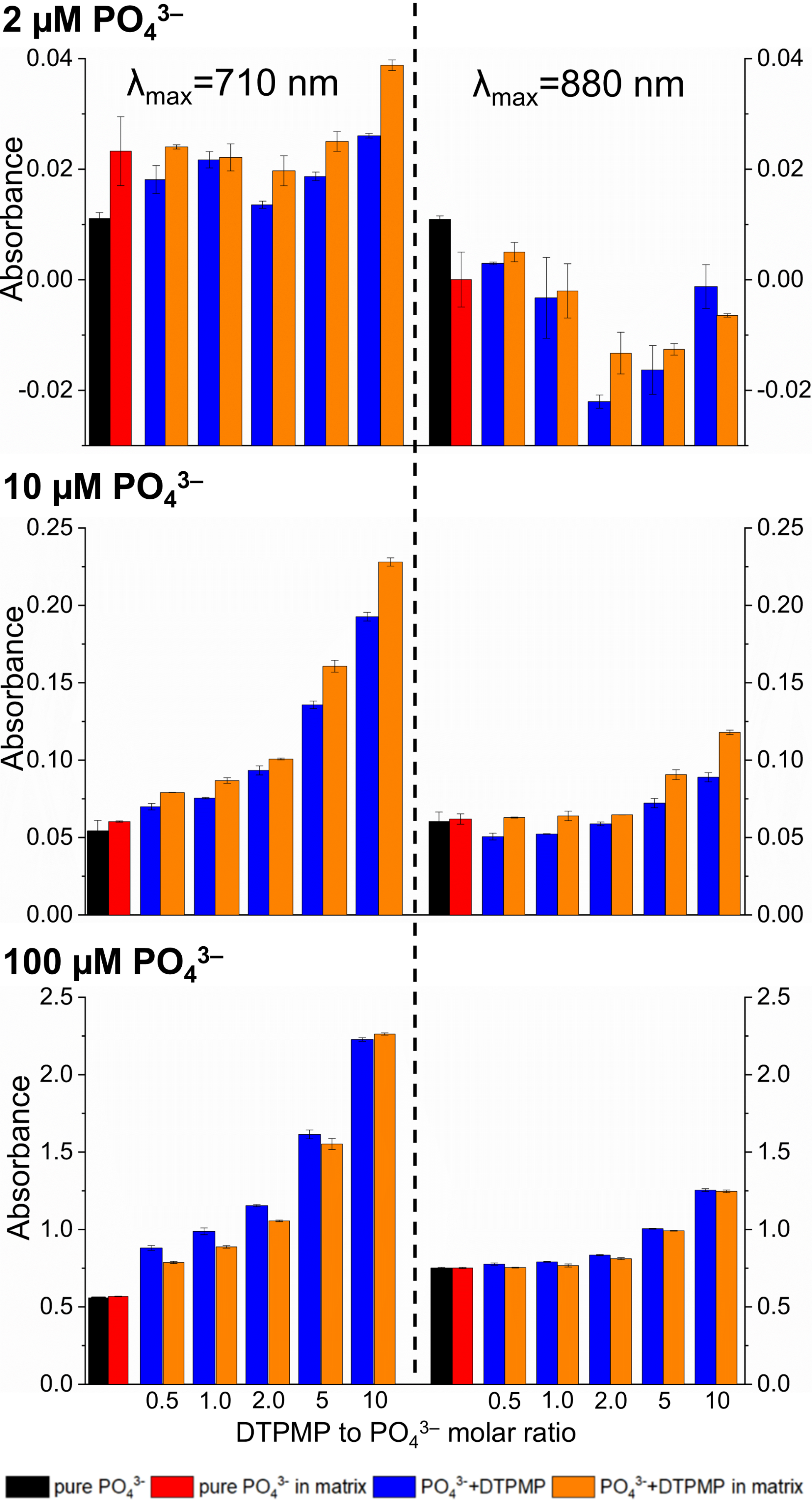
**

***Fig. S6*** *Absorbance at λ_max_=710 nm (left) and 880 nm (right) for fixed PO_4_^3‒^ concentration of 2 µM, 10 µM, and 100 µM, with varying DTPMP to PO_4_^3‒^ molar ratios, in ultrapure water and matrix, using the MB_Tü_ method. Columns share the same x-axis, and rows share the same y-axis. Error bars represent the standard deviation of triplicate experiments.*

**References**

1. International Organization for Standardization. Water quality — Determination of phosphorus — Ammonium molybdate spectrometric method (ISO Standard No. 6878:2004). 2004.

2. U.S. Environmental Protection Agency. Method 365.3: Phosphorous, All Forms (Colorimetric, Ascorbic Acid, Two Reagent). 1978.

3. American Public Health Association. Standard methods for the examination of water and wastewater (23rd ed.). Washington DC: APHA Press; 2017.

4. Laskov C, Herzog C, Lewandowski J, Hupfer M. Miniaturized photometrical methods for the rapid analysis of phosphate, ammonium, ferrous iron, and sulfate in pore water of freshwater sediments. Limnology & Ocean Methods. 2007 Jan;5(1):63–71.

5. DeSilva MA, Shanaiah N, Nagana Gowda GA, Rosa‐Pérez K, Hanson BA, Raftery D. Application of ^31^ P NMR spectroscopy and chemical derivatization for metabolite profiling of lipophilic compounds in human serum. Magnetic Reson in Chemistry [Internet]. 2009 Dec [cited 2024 Oct 30];47(S1). Available from: https://analyticalsciencejournals.onlinelibrary.wiley.com/doi/10.1002/mrc.2480
